# Spatio-temporal genetic structure of *Anopheles gambiae* in the Northwestern Lake Victoria Basin, Uganda: implications for genetic control trials in malaria endemic regions

**DOI:** 10.1186/s13071-018-2826-4

**Published:** 2018-04-16

**Authors:** Martin Lukindu, Christina M. Bergey, Rachel M. Wiltshire, Scott T. Small, Brian P. Bourke, Jonathan K. Kayondo, Nora J. Besansky

**Affiliations:** 1 Department of Biological Sciences and Eck Institute for Global Health, University of Notre Dame, Notre Dame, IN 46556 USA; 2 Department of Entomology, Uganda Virus Research Institute (UVRI), Entebbe, Uganda

**Keywords:** *Anopheles gambiae*, Gene flow, Lacustrine islands, Malaria, Mitochondrial DNA, Population genetic structure, Uganda

## Abstract

**Background:**

Understanding population genetic structure in the malaria vector *Anopheles gambiae* (*s.s*.) is crucial to inform genetic control and manage insecticide resistance. Unfortunately, species characteristics such as high nucleotide diversity, large effective population size, recent range expansion, and high dispersal ability complicate the inference of genetic structure across its range in sub-Saharan Africa. The ocean, along with the Great Rift Valley, is one of the few recognized barriers to gene flow in this species, but the effect of inland lakes, which could be useful sites for initial testing of genetic control strategies, is relatively understudied. Here we examine Lake Victoria as a barrier between the Ugandan mainland and the Ssese Islands, which lie up to 60 km offshore. We use mitochondrial DNA (mtDNA) from populations sampled in 2002, 2012 and 2015, and perform Bayesian cluster analysis on mtDNA combined with microsatellite data previously generated from the same 2002 mosquito DNA samples.

**Results:**

Hierarchical analysis of molecular variance and Bayesian clustering support significant differentiation between the mainland and lacustrine islands. In an mtDNA haplotype network constructed from this and previous data, haplotypes are shared even between localities separated by the Rift Valley, a result that more likely reflects retention of shared ancestral polymorphism than contemporary gene flow.

**Conclusions:**

The relative genetic isolation of *An. gambiae* on the Ssese Islands, their small size, level terrain and ease of access from the mainland, the relative simplicity of the vectorial system, and the prevalence of malaria, are all attributes that recommend these islands as possible sites for the testing of genetic control strategies.

## Background

*Anopheles gambiae* (*s.s.*) (formerly *An. gambiae* S-form) is a principal vector of malaria in sub-Saharan Africa, where 91% of an estimated 445,000 malaria deaths worldwide occurred in 2016 [1]. This species is distributed broadly across most of tropical Africa, including its offshore islands, occupying a diversity of ecological settings but almost invariably in association with rural or peri-urban human populations [2–4]. Improved understanding of the population connectivity of *An. gambiae* (*s.s*.) (henceforth *An. gambiae*) is of critical importance, given its relevance both to insecticide resistance management and to potential future application of genetic control strategies. However, *An. gambiae* is characterized by very large effective population sizes, high fecundity and dispersal potential, and extremely high genetic diversity [5–9]. These factors mitigate or prevent spatial genetic differentiation, even between populations that are almost completely isolated demographically, in the absence of any physical barriers to dispersal [10–12]. Few physical barriers to *An. gambiae* dispersal are known across continental Africa and consequently very shallow spatial population genetic structure exists continent-wide, with the notable exception of populations separated by the Rift Valley [9, 13–17]. Based on microsatellite markers, the magnitude of genetic differentiation (*F*_ST_) between populations on opposite sides of the continent (~6,000 km apart) is ~0.03, while the corresponding value between populations on either side of the Rift Valley (~400–500 km) is ~0.1 [14–17].

Not surprisingly, the ocean represents a barrier to *An. gambiae* gene flow between mainland Africa and its offshore islands [18–21]. Although passive migration through the upper airstream or hitchhiking with human transport are possible [8, 22], indirect genetic evidence supports substantial isolation imposed by the ocean, given sufficient distance between the continent and an oceanic island. Based on microsatellites or small-scale single nucleotide polymorphism (SNP) arrays, differentiation between mainland and oceanic islands is negligible or slight at distances of 50–100 km (*F*_ST_: 0–0.02) ([19, 20], but see [23]) but progressively stronger at distances of 300–400 km (*F*_ST_: 0–0.05) [21] and 600–800 km (*F*_ST_: ~ 0.2) [20].

In contrast to the ocean barriers, the role of large mainland lakes as barriers to gene flow in *An. gambiae* has received less attention. Lake Victoria, which borders Kenya, Uganda and Tanzania, is the largest lake in Africa, with an area of approximately 68,800 km^2^. Two previous studies have examined the level of genetic differentiation between mainland and island sites in Lake Victoria. Working in the Suba District of western Kenya at the northeast edge of Lake Victoria, Chen et al. [24] sampled *An. gambiae* on island and mainland sites separated by relatively short distances (~5–35 km), with one of the islands connected to the mainland by a causeway. Overall, they observed extremely low but significant genetic differentiation at six microsatellite loci between island and mainland populations (global *F*_ST_ = 0.003). The only other investigation, to our knowledge, is that of Kayondo et al. [25], who sampled *An. gambiae* from four Ugandan islands in the northwestern part of Lake Victoria, separated from two sampled mainland sites by greater distances (~22–111 km) than those of the Kenyan study. Across the 13 microsatellite loci beyond the neighborhood of chromosomal inversions, the overall level of lacustrine island-mainland differentiation was both significant and relatively high (mean pairwise *F*_ST_ = 0.06; from Table 6 of [25]), and larger than the value estimated for oceanic island-mainland differentiation at a distance of 300–400 km [21].

To improve our understanding of freshwater lakes as barriers to gene flow in *An. gambiae*, we expand on the study of Kayondo et al. [25] by adding a new marker system, the non-recombining maternally inherited mitochondrial DNA (mtDNA) genome, increasing the number of mainland and island sampling locations, and introducing a temporal dimension through repeated mtDNA sampling in 2002, 2012 and 2015. In addition, we revisit the microsatellite data of Kayondo et al. [25] after stringent filtering, and apply Bayesian clustering for inference of population structure. As expected, we find temporal variation among islands and moderate genetic structure between islands and mainland. To address the significance of our results with respect to the confinement needed for the phased testing of genetically modified mosquitoes (especially gene drives) [26, 27], we consider these findings in light of the life history traits of *An. gambiae* and a synthesis of published estimates of its geographical population structure. We conclude that the degree of lacustrine island isolation from mainland Uganda may be sufficient to consider one or more of these islands as possible locations for testing the safety and efficacy of genetic control strategies.

## Methods

### Population sampling

**Fig. 1:**
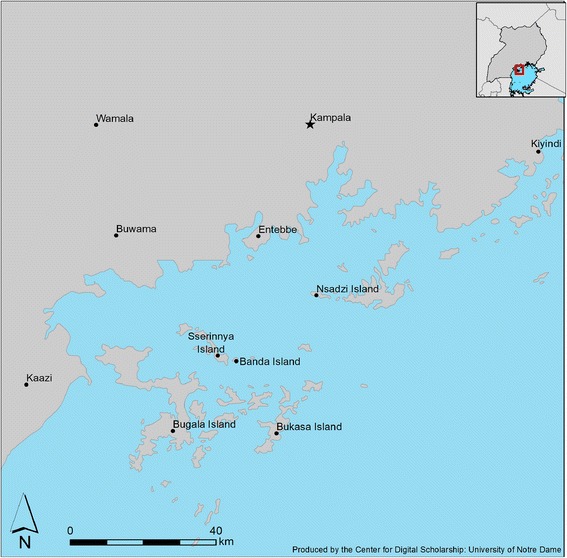
Map showing location of sampling sites in the Lake Victoria Basin of Uganda. More detailed information is provided in Table 1

The study area lies within the northwestern Lake Victoria Basin in southern Uganda (Fig. 1). This part of Lake Victoria contains an archipelago of 84 islands, known as the Ssese Islands, whose total land mass covers 432 km^2^. Of the 64 inhabited islands in this archipelago, the largest is Bugala (296 km^2^) which is connected by ship and ferry service to the Ugandan mainland. The total human population on the Ssese Islands is estimated at approximately 54,000 [28]. Urbanization is low (~8%), and livelihood depends mainly on fishing and oil palm production. In November 2001 - February 2002, Kayondo et al. [24] sampled *An. gambiae* mosquitoes from two mainland localities near the shores of Lake Victoria (Wamala and Entebbe) and four islands in the Ssese archipelago (Bugala, Bukasa, Nsadzi and Sserinya). Genomic DNA archived from the 2001–2002 collection (henceforth referred to as the ‘2002’ collection for simplicity) was available to us for the present study (Table 1). Building on that study, we temporally sampled most of these same localities in subsequent years, in July-October 2012 and again in May-June 2015, also adding one additional Ssese island (the tiny island of Banda) and three additional mainland localities (Buwama, Kaazi and Kiyindi) (Table 1, Fig. 1). Buwama is a small village located in central Uganda along the Masaka-Kampala highway. Though the main activity in Buwama is subsistence agriculture, small scale trade is carried out, especially at the fringes of the busy highway. Kaazi village is situated close to the shores Lake Nabugabo, a satellite lake of Lake Victoria. Most of the population is involved in subsistence agriculture. Kiyindi is a landing site on the shores of Lake Victoria, where most of the local population is directly or indirectly involved in the fishing industry.

**Table 1:** Mitochondrial samples and sequence polymorphism summary statistics

Site (CODE)	Coordinates (Lat, Long)	Year (MG/*nad*5)	*n*	Π ± SD	θ	Hd ± SD	*D*
Wamala^a^ (WML)	0.3459, 32.0541	2015 (MG)	11	0.004 ± 0.0003	0.005	0.982 ± 0.046	-1.411
		2015 (*nad*5)	11	0.006 ± 0.0013	0.008	0.945 ± 0.054	-0.912
		2012 (*nad*5)	27	0.004 ± 0.0006	0.006	0.855 ± 0.060	-1.232
		2002 (*nad*5)	17	0.005 ± 0.0010	0.009	0.875 ± 0.070	-1.540
Buwama^a^ (BWM)	0.0631, 32.1031	2015 (MG)	11	0.003 ± 0.0002	0.005	1.000 ± 0.039	-1.615
		2015 (*nad*5)	11	0.007 ± 0.0011	0.008	0.964 ± 0.051	-0.951
		2012 (*nad*5)	–	–	–	–	–
		2002 (*nad*5)	–	–	–	–	–
Kaazi^a^ (KZI)	-0.3183, 31.8818	2015 (MG)	11	0.004 ± 0.0004	0.005	0.982 ± 0.046	-1.445
		2015 (*nad*5)	11	0.007 ± 0.0010	0.008	0.945 ± 0.066	-0.683
		2012 (*nad*5)	–	–	–	–	–
		2002 (*nad*5)	–	–	–	–	–
Kiyindi^a^ (KYD)	0.2776, 33.1459	2015 (MG)	10	0.003 ± 0.0002	0.005	1.000 ± 0.045	-1.494
		2015 (*nad*5)	10	0.008 ± 0.0013	0.010	0.978 ± 0.054	-1.093
		2012 (*nad*5)	–	–	–	–	–
		2002 (*nad*5)	–	–	–	–	–
Entebbe^a^ (EBB)	0.0613, 32.4544	2015 (*MG*)	–	–	–	–	–
		2015 (*nad*5)	–	–	–	–	–
		2012 (*nad*5)	32	0.005 ± 0.0007	0.008	0.935 ± 0.021	-0.910
		2002 (*nad*5)	22	0.005 ± 0.0006	0.008	0.952 ± 0.037	-1.193
Bukasa (BKS)	-0.4428, 32.4994	2015 (MG)	11	0.003 ± 0.0003	0.003	0.927 ± 0.066	-0.322
		2015 (*nad*5)	11	0.006 ± 0.0009	0.005	0.855 ± 0.085	0.710
		2012 (*nad*5)	32	0.006 ± 0.0006	0.006	0.885 ± 0.039	-0.737
		2002 (*nad*5)	28	0.005 ± 0.0005	0.006	0.934 ± 0.027	-0.708
Bugala (BGL)	-0.4369, 32.2436	2015 (MG)	23	0.003 ± 0.0002	0.006	0.968 ± 0.026	-1.827^b^
		2015 (*nad*5)	23	0.005 ± 0.0005	0.008	0.945 ± 0.027	-1.128
		2012 (*nad*5)	32	0.006 ± 0.0006	0.008	0.925 ± 0.028	-0.860
		2002 (*nad*5)	26	0.005 ± 0.0006	0.007	0.942 ± 0.022	-1.017
Nsadzi (NSZ)	-0.0901, 32.5979	2015 (MG)	12	0.003 ± 0.0003	0.003	0.833 ± 0.100	-0.433
		2015 (*nad*5)	12	0.008 ± 0.0008	0.007	0.818 ± 0.100	0.391
		2012 (*nad*5)	37	0.006 ± 0.0005	0.005	0.875 ± 0.028	0.470
		2002 (*nad*5)	30	0.006 ± 0.0007	0.006	0.915 ± 0.023	-0.255
Banda (BDA)	-0.2581, 32.4003	2015 (MG)	11	0.003 ± 0.0028	0.004	0.927 ± 0.066	-0.476
		2015 (*nad*5)	11	0.006 ± 0.0006	0.005	0.891 ± 0.092	0.269
		2012 (*nad*5)	–	–	–	–	–
		2002 (*nad*5)	–	–	–	–	–
Sserinnya (SYR)	-0.2445, 32.3543	2015 (MG)	14	0.006 ± 0.0004	0.004	0.890 ± 0.060	-0.855
		2015 (*nad*5)	14	0.004 ± 0.0003	0.005	0.923 ± 0.050	0.290
		2012 (*nad*5)	–	–	–	–	–
		2002 (*nad*5)	26	0.006 ± 0.0007	0.008	0.954 ± 0.022	-1.033

^a^Mainland sites

^b^*P* < 0.05

*Abbreviations*: *MG* protein-coding mitogenome, *nad*5 fragment of mtDNA *nad*5 gene, *n* number of mosquitoes in sample, π nucleotide diversity per site, *SD* standard deviation, θ theta (per site) from S (number of segregating sites), *Hd* haplotype (gene) diversity, *D* Tajima’s D

### Mosquito collection, identification and preservation

Indoor resting adult mosquitoes were collected by aspiration or insecticide spray catch. Morphological identification of *An. gambiae* (*s.l*.) was performed with the aid of a taxonomic key [29]. Mosquitoes were then preserved individually with a desiccant and transported to the University of Notre Dame, USA, where females were molecularly identified to species using a ribosomal DNA-based PCR assay [30] performed directly on one leg or wing. The only member of the *An. gambiae* (*s.l*.) species complex identified in our collections other than *An. gambiae* (*s.s*.) was *An. arabiensis*, in very low numbers. Thus, only *An. gambiae* (*s.s*.) was further analyzed in this study.

### Mitochondrial DNA sequencing and analysis

Individual mosquitoes collected in 2015 were subject to whole genome sequencing on the Illumina HiSeq 2500 with 100 paired end cycles as part of a separate population genomic study. Here, the mitogenomes of these individual mosquitoes were assembled from the subset of Illumina reads aligning to the complete mitochondrial genome of *An. gambiae* ([31], GenBank: L20934). To ensure that reads derived from a circular mitochondrial genome were correctly paired in mapping if they overlapped the origin of replication, the reference mitochondrial genome was first modified by appending nucleotides from position 1 to 5000 onto the end [32]. Whole genome Illumina reads from individual mosquitoes were mapped to this modified mitochondrial reference using bowtie2 v2.2.9 [33] with the following options: --no-unal -X 700 -R 5 -N 1 -L 12 -D 25 -i S,2,.25. All reads that were not properly paired were removed to mitigate against the inclusion of nuclear-integrated mitochondrial sequences (numts). The remaining set of mapped reads were then *de novo* assembled using the default assembler and default options available in Geneious v10.0.3 [34]. Resulting contigs were aligned to the unmodified *An. gambiae* mitochondrial genome and scaffolded, introducing “N” between *de novo* assembled contigs as needed. Finally, *de novo* assembled mitochondrial genomes were annotated by transferring annotations from the *An. gambiae* genome after alignment with MAFFT [35]. All annotations were examined for premature stop codons and frame-shifts using Geneious. The 114 assembled mitogenomes have been submitted to GenBank under accessions MG753657–MG753770. Prior to analysis, the A+T-rich origin of replication and tRNAs were removed, thus measures of diversity and differentiation presented below are based solely on protein-coding mtDNA sequences.

For the 2002 and 2012 collections, only a portion of the mitochondrial *nad*5 gene was sequenced, using genomic DNA archived from Kayondo et al. [25] or, in the case of 2012 specimens, extracted using a CTAB protocol [36]. Amplification of the *nad*5 region was performed by PCR in a 25 μl reaction mixture containing primers 19CL (5'-CTT CCA CCA ATT ACT ATA ACA G-3') and DMP3A (5’-AGG ATG AGA TGG CTT AGG TT-3') [37]. The PCR amplification protocol consisted of a 2 min denaturation at 95 °C and 35 cycles at 94 °C for 30 s, 54 °C for 30 s and 72 °C for 1 min each, followed by a 5 min extension at 72 °C, and then a holding temperature of 4 °C. Quality of amplification was assessed by 1% agarose gel electrophoresis of 5 μl aliquots. Amplicons were purified prior to sequencing with the ExoSAP-IT PCR purification kit (USB Corporation, OH, USA). Sanger sequencing of both strands was conducted on an Applied Biosystems 96-capillary 3730xl DNA Analyzer at the Genomics and Bioinformatics core facility, University of Notre Dame. Raw sequences were visually inspected and edited using Geneious v10.0.3 [34]. Corrected forward and reverse sequences were merged into a consensus, and the resulting set of consensus sequences were submitted to GenBank under accessions MG744715-MG745137. These sequences were aligned in MEGA v7.0.14 [38] using MUSCLE. Sequences were scanned for stop codons in the translation frame using the translation tool and the invertebrate mitochondrial code.

Measures of DNA sequence polymorphism for each population were computed in DnaSP v5.10.01 [39]. Sequence differentiation was calculated in Arlequin 3.5 [40] using an analog of *F*_ST_ (Φ_ST_) that takes pairwise differences between DNA haplotypes into account; significance was determined based on 1000 random permutations. In addition, we performed hierarchical Analysis of Molecular Variance (AMOVA) [41] in Arlequin 3.5 to assess spatiotemporal differentiation in the Ssese Islands, based on the three islands for which we had three years of data (2002, 2012 and 2015; Table 1). We tested alternative scenarios that differed in how the data were nested: years clustered within islands, and islands clustered within years. A mtDNA *nad*5 haplotype network was constructed using the TCS inference method [42] implemented in POPART [43].

### Microsatellite analysis

Starting with the genotypes at 17 microsatellite loci obtained by Kayondo et al. [25] from year 1 (2001–2002) of that study, we performed pairwise tests of linkage disequilibrium (LD) using an exact test based on a Markov chain method, as implemented in GENEPOP 4.2 [44], and found no pairs in LD that were physically linked. We pruned loci that mapped within or near the breakpoints of inversions 2La and 2Rb (H79, 22C1, MBP1A, MBP1B), and those whose accurate genotyping was deemed problematic, due to unexpected allele size distributions. We retained for analysis 9 loci for which we had high confidence in genotyping accuracy: (AgX)H145CD, (AgX)H99, (AgX)ID1, (Ag2)H117, (Ag3)H93, (Ag3)H158, (Ag3)33C1, (Ag3)H817 and (Ag3)H577. These data are available from the Dryad Digital Repository (10.5061/dryad.7p177s7). MICRO-CHECKER 2.2.3 [45] found evidence of potential null alleles at five of these loci in up to three population samples (null allele frequency of H117: 14.5–27.7%; H145CD: 11–12%; 33C1: 12–17.5%; H158: 9.5%; H93: 9–12.1%), and consistent with the previous analyses of Kayondo et al. [25], predicted that all populations were in Hardy-Weinberg equilibrium after accounting for the contribution of suspected null alleles to heterozygote deficits. Previous simulation and empirical studies have suggested that null alleles at these frequencies should have little effect on our assessment of population structure [46, 47].

Using the set of genotypes determined at these 9 loci, combined with the mtDNA *nad*5 sequence from each corresponding mosquito, we analyzed the distribution of genetic variants among the two mainland and four island locations sampled in 2002 using the iterative Bayesian clustering approach implemented in STRUCTURE v2.3.4 [48]. Under the expectation of shared recent ancestry and admixed ancestry, respectively [9, 18], we ran STRUCTURE under the ‘correlated’ allele frequency and ‘admixture’ ancestry models. We included sampling location as a prior (LOCPRIOR), as this has been shown to improve inference when genetic data have a weak signal, without finding population structure where it does not exist [49]. After an initial 200,000 'burn-in' period, each run included 500,000 Markov Chain Monte Carlo (MCMC) iterations. Ten independent replicate runs were performed for each value of K (assumed populations or genetic groups), from 1 to 6. Post-processing of STRUCTURE results, including choosing the optimal K value and producing the graphical output, was implemented using CLUMPAK [50].

## Results

Complete mtDNA genomes were assembled from 114 individually sequenced *An. gambiae* collected in 2015 from five Ssese Island sites and four mainland sites near the shores of Lake Victoria (Table 1, Fig. 1). We analyzed the protein-coding bases of the mitogenome (14,844; 97%), and compared these results to those obtained from a 525 bp fragment of the mtDNA *nad*5 gene amplified and sequenced from archived *An. gambiae* collections from the same or nearby localities made in 2002 and 2012. Across all 423 sequences, we found no evidence of stop codons and only two non-synonymous substitutions in the *nad*5 gene.

### Mitochondrial haplotype diversity is high but reduced on islands

Across the protein-coding mitogenome, mean nucleotide diversity was 0.0033 in the 2015 sample of 114 mosquitoes, while the mean nucleotide diversity for just the *nad*5 gene fragment in this sample was higher at 0.0065, comparable to the mean estimates for this fragment in the 149 mosquitoes in the 2002 sample (0.0052) and the 160 mosquitoes in the 2012 sample (0.0054). Haplotype diversity was generally very high across space and time, but it is evident from the 2015 samples (which include the most data for comparison) that mean haplotype diversity is higher on the mainland than on the islands (0.991 *vs* 0.909 for protein-coding mitogenome, *P* = 0.008; 0.958 *vs* 0.886 for *nad*5, *P* = 0.016). Consistent with previous findings for this species, haplotype diversity is driven by an abundance of singleton haplotypes. Of the 97 haplotypes in the total set of 423 sequences, 65 (67%) were singleton haplotypes (observed only once) and another 6 were doubletons.

### Mitochondrial differentiation suggests genetic structure between mainland and islands

Using hierarchical analysis of molecular variance (AMOVA) based on the protein-coding mitogenome, we estimated global *F*_ST_ among population samples from the mainland and similarly, among population samples from the Ssese Islands. Whereas *F*_ST_ was not significantly different from zero on the mainland, it was moderate and statistically significant for the islands (0.056; *P* < 0.001; Table 2). Comparable results were obtained when this approach was applied to only the *nad*5 gene fragment, regardless of sampling period (Table 2).

**Table 2:** Summary of spatial population genetic differentiation (global *F*_ST_) inferred from mtDNA

Year (MG/*nad*5)	Among mainland	Among islands	Island-Mainland
2015 (MG)	-0.004^ns^	0.056^***^	0.059^***^
2015 (*nad*5)	-0.012^ns^	0.071^**^	0.071^**^
2012 (*nad*5)	0.025^ns^	0.046^**^	0.065^***^
2002 (*nad*5)	-0.007^ns^	0.011^ns^	0.077^***^

*Abbreviations*: *MG* protein-coding mitogenome, *nad*5 fragment of mtDNA *nad*5 gene, *ns* not significant

**P < 0.01, ***P < 0.001

**Fig. 2:**
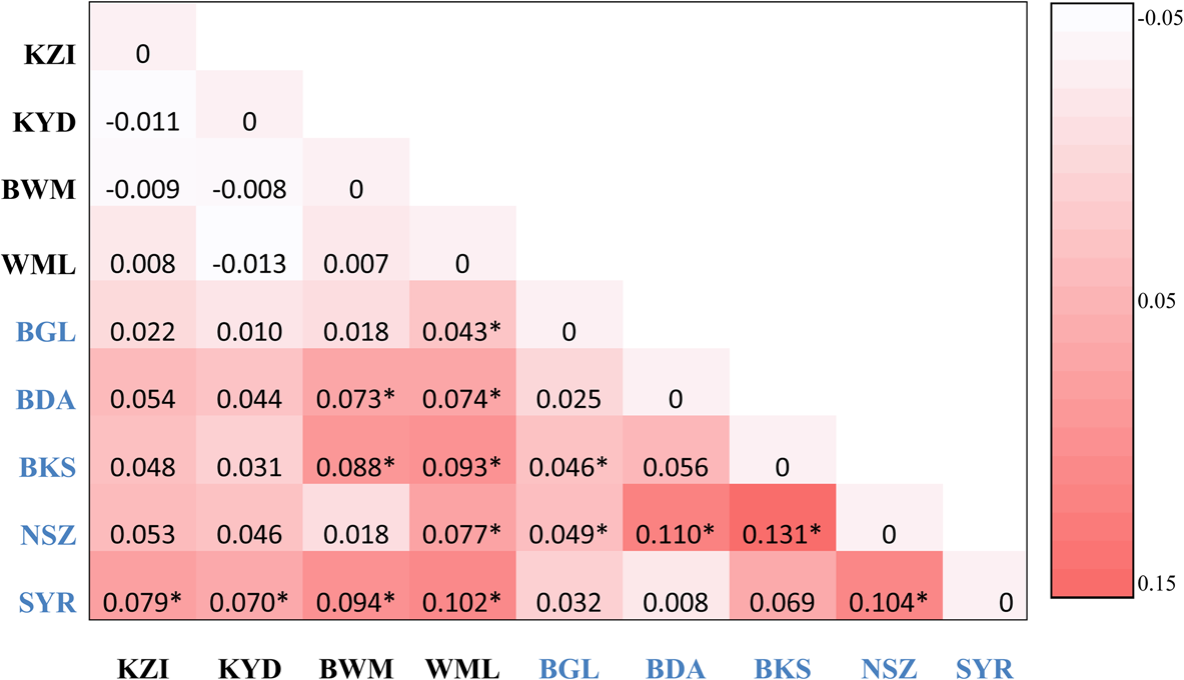
Heatmap of pairwise *F*_ST_ values inferred from the protein-coding mitogenome. Values statistically different from zero (*P* < 0.05) are indicated with an asterisk. Population codes from islands are in blue font

We also used hierarchical AMOVA to infer population genetic structure between mainland and island populations, testing this for the protein-coding mitogenome data from 2015 and the *nad*5 data from each the three sampling periods (Table 2). Again, *F*_ST_ estimates were comparable between data sets, statistically significant, and similar in magnitude to inter-island *F*_ST_ values, ranging from 0.06 to 0.08. Illustrating these results graphically, Fig. 2 represents a matrix of pairwise *F*_ST_ values from the mitogenome data of 2015, color-coded such that increasing saturation reflects larger values. Notably, *F*_ST_ values tend to be highest between mainland and island or among island samples, and are not significant among mainland samples.

### Bayesian cluster analysis also supports genetic structure between mainland and islands

**Fig. 3:**
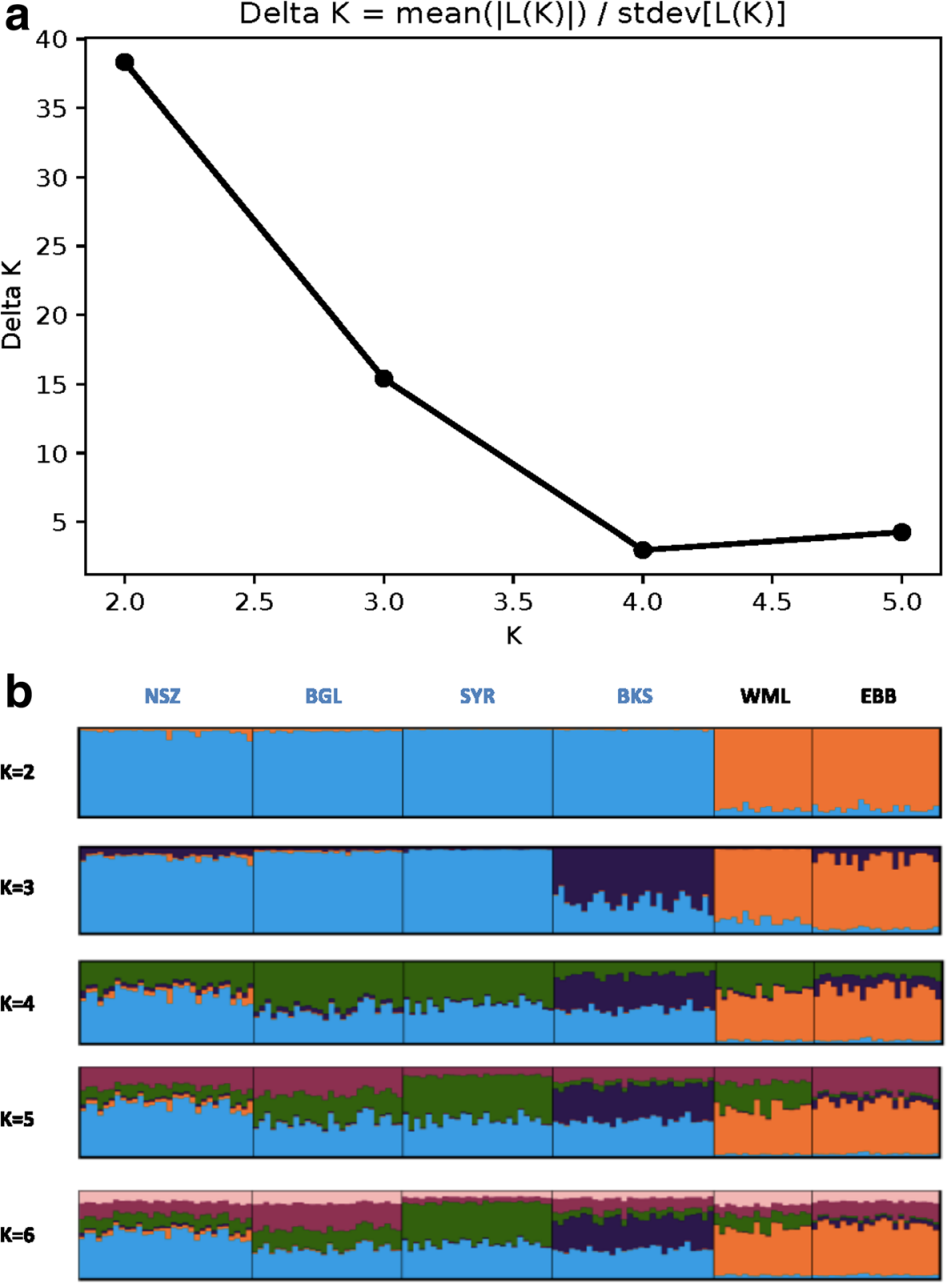
Bayesian clustering analysis of combined 2002 microsatellite and mtDNA data from the same mosquito specimens. **a** The optimal number of clusters (*K*) was two based on the method of Evanno et al. [55]. **b** Bar plots with individual mosquitoes represented as vertical bars colored in proportion to their assignment to clusters inferred at each value of *K* (from 2 to 6). Individuals from the same population sample are grouped, with groups bounded by vertical black lines. Population labels are in blue font for islands and black font for mainland samples

Our mtDNA data from 2002 were generated from DNA archived by Kayondo et al. [25], and hence derive from the same specimens employed in that study (where they are referred to as Year 1, 2001–2002). We combined the *nad*5 mtDNA sequences together with the corresponding microsatellite data from nine loci (after pruning the latter of loci near chromosomal inversions and those whose genotyping was less confident; see Methods). Bayesian cluster analysis supported K = 2 as the optimal number of clusters (Fig. 3), with one consisting of the two mainland samples, and the other containing the four island samples. These results did not materially change when the *nad*5 data were omitted and only microsatellite data were subjected to cluster analysis (data not shown).

### Small but significant temporal variation on islands and mainland

Three islands were repeatedly sampled in all three time periods, as was one mainland site (Wamala). We used the *nad*5 sequences from these population samples to assess the degree of temporal variation, with the expectation that temporal variation on islands would be significant given the smaller effective population size of *An. gambiae* on these islands relative to mainland populations [25]. Two alternative scenarios were tested by hierarchical AMOVA for the island sites (Table 3). In the first, years were clustered within islands; in the second, islands were clustered within years. There was a small yet significant amount of variation (~5%) explained by sampling the same island locality at different time periods, as predicted. With islands clustered within years, differences between sampling periods were swamped by among-island differences for each period. Unexpectedly, we also observed significant temporal variation in Wamala (~5%; *F*_ST_ = 0.05, *P* = 0.01)

**Table 3:** Hierarchical AMOVA of mtDNA *nad*5 variation within and among three island populations and three sampling periods (years)^a^

Scenario	Hierarchical level	Variation (%)	Φ	*P* ^b^
Years Clustered Within Islands	Among islands	0.62	Φ_CT_ 0.0062	0.254
	Among years within islands	4.55	Φ_SC_ 0.0458	< 0.001
	Within subpopulations	94.83	Φ_ST_ 0.0517	< 0.001
Islands Clustered Within Years	Among years	0.11	Φ_CT_ 0.0011	0.285
	Among islands within years	4.94	Φ_SC_ 0.0495	< 0.001
	Within subpopulations	94.94	Φ_ST_ 0.0506	< 0.001

^a^Islands of Bugala, Bukasa and Nsadzi, in 2002, 2012 and 2015.

^b^Probability of obtaining a more extreme Φ value by chance alone, determined by 1000 random permutations in Arlequin 3.5

## Discussion

Using either whole protein-coding mitogenomes or a relatively small fragment of the mtDNA *nad*5 gene, we obtained evidence consistent with restricted connectivity between the Ssese Islands in Lake Victoria and the Ugandan mainland, despite the fact that the islands sampled were within 60 km of the mainland. Our results corroborate the previous microsatellite study of Kayondo et al. [25] and a more recent reduced-representation genome sequencing effort (RM Wiltshire, CM Bergey, JK Kayondo, J Birungi, LG Mukwaya, SJ Emrich, NJ Besansky, and FH Collins, unpublished observations), both of which concluded that significant variation in allele frequency was likely the result of the combined effects of reduced population connectivity and smaller effective population sizes on the islands. In agreement with this finding, we observed slight but significant allele frequency variation between sampling periods on a given island and on the mainland site of Wamala, consistent with the effects of genetic drift and possibly seasonal or other bottlenecks. Bayesian clustering analysis to detect genetic discontinuities complemented the previous *F*_ST_-based approaches, revealing separate mainland and island clusters that may reflect shared ancestry among island populations stemming from a single initial historical colonization event from the mainland.

**Fig. 4:**
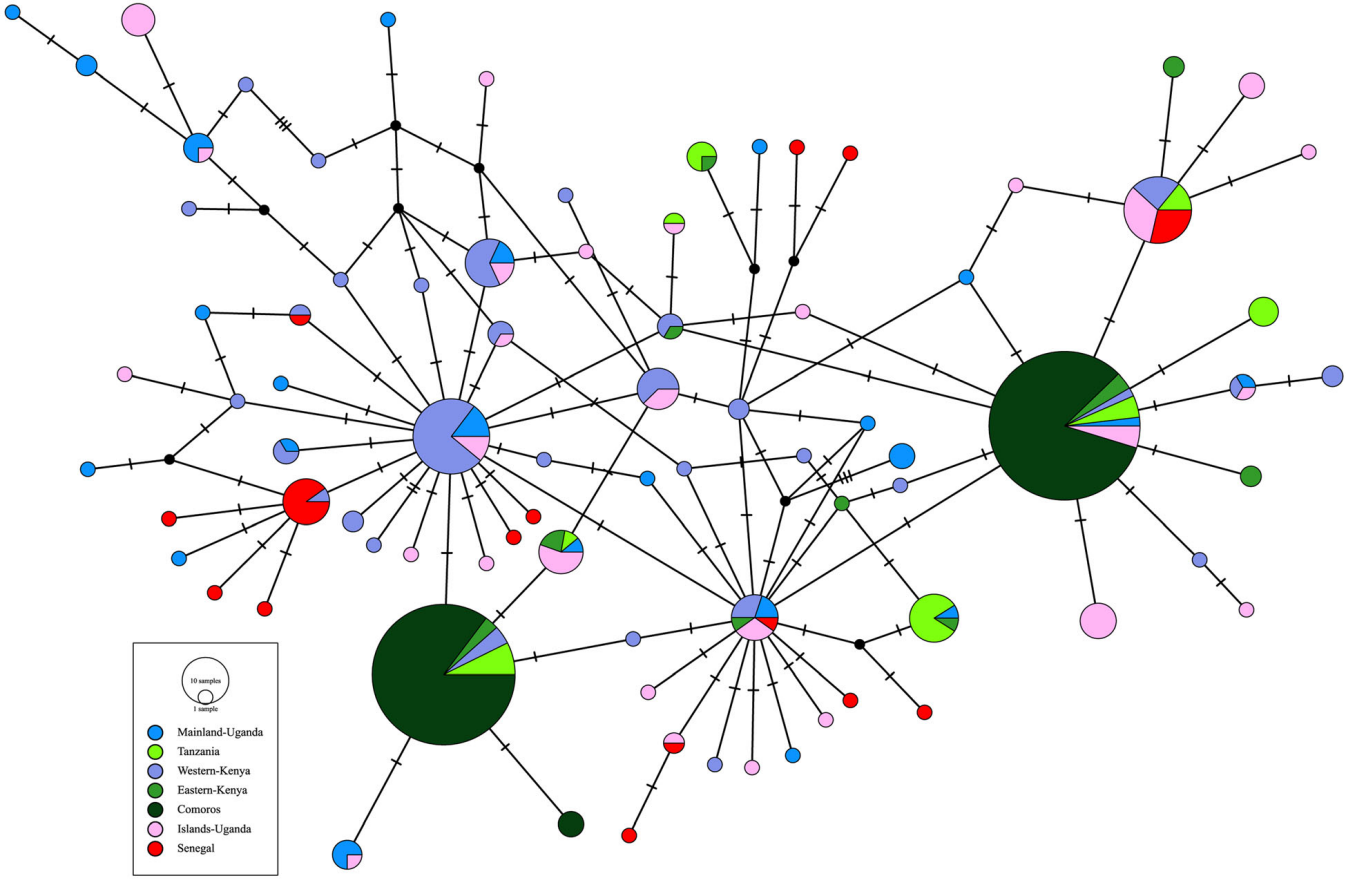
Mitochondrial DNA *nad*5 haplotype TCS network based on the 2015 sample from this study and additional *nad*5 sequences sampled from previous studies in Senegal, Kenya, Tanzania, and the Comoros [20, 37, 56] downloaded from GenBank. Each observed haplotype is indicated by a filled circle, sized according to its frequency and colored according to the location(s) where it was sampled. Inferred (unobserved) haplotypes are indicated by small black circles. Haplotype relationships are indicated by lines; mutational steps between haplotypes are represented by the number of hatches

How much isolation actually exists in *An. gambiae*? It is unlikely that populations follow the equilibrium assumptions of Wright’s island model [51]. Multiple studies support the notion of a relatively recent population size and range expansion by this vector [6, 18], frequent local extinction and long distance recolonization [8], and a very large long-term effective population size [5]. As is now widely acknowledged, violation of the often unrealistic assumptions of evolutionary equilibrium implies that there is no simple equation between population genetic differentiation (e.g. *F*_ST_ and its analogs) and migration rate [52, 53]. Large effective population size alone limits *F*_ST_, because of shared ancestral polymorphism and not necessarily recurrent (contemporary) gene flow [10, 53]. The extensive mtDNA haplotype sharing across long geographical distances (e.g. 6,000–7000 km of continental Africa) has been noted previously [18, 20, 37], but because this issue bears on the interpretation of the relatively weak level of differentiation (*F*_ST_) observed between islands in Lake Victoria and the Ugandan mainland, we constructed an *nad*5 haplotype network using haplotypes from the current study as well as those detected previously in Senegal (West Africa), on the East African mainland, and the Comoros. With the caveat that our sequences were shorter than those deposited into GenBank, requiring us to trim down to the shortest common length (525 bp) and thus reduce the number of haplotypes relative to those reported in the original studies, Fig. 4 shows a level of haplotype sharing (notably, between Senegal and coastal Kenyan or Tanzanian locations separated by the Great Rift Valley) that is more reasonably explained by recent common ancestry than by contemporary gene flow. We think it likely that the connectivity between Ssese Island and Uganda mainland populations is low, but more firmly disentangling and quantifying the confounding forces will require additional data and, if feasible, direct estimates of dispersal.

To place our results in the framework of other studies of genetic differentiation in *An. gambiae*, we summarized *F*_ST_ values estimated at different ranges of geographical distances, drawing from this and previous studies and partitioning the data by marker class (mtDNA, microsatellites and SNPs) and type of contrast (among mainland sites, or between mainland and either oceanic or lacustrine islands). The results, shown in Table 4, emphasize the general importance of water in imposing spatial population genetic structure in this species, but also highlight important parallels between oceanic and freshwater barriers, a point that may be underappreciated. Here we find that levels of population differentiation between mainland and island appear comparable at similar distances over either ocean or lake barriers.

**Table 4:** Geographical population structure indicated by *F*_ST_. Mean of pairwise *F*_ST_ values reported by cited studies, unless noted

Contrast	Distance (km)	mtDNA *nad*5	Microsatellites	SNP/SNP Array
Mainland	5–100	0.017 [37]	0.0039^a^–0.013^b^	–
	200–800	0.032 [37]	0.0064^a^–0.031^b^	–
	1000–7000	0.085 [37]	0.0161^a^–0.039^b^	0.023^g^
Main-Oceanic	50–100	–	0.0238^c^	0–0.016^h^
	300–400	–	0.050^d^	–
	600–800	–	–	0.20–0.25^h^
Main-Lacustrine	2–20	–	0.003^e^	–
	25–100	0.059–0.077	0.070^f^ (0.062)	–

^a^Average across 9 loci from [16], only comparisons within NW and SE subdivisions for *An. gambiae* (S-form)

^b^Average across 13 chromosome-3 loci from [17], only comparisons within *An. gambiae* (S-form) excluding Mozambique

^c^Average across 8 chromosome-3 loci from [19], only comparison within *An. gambiae* (S-form) between Tiko (mainland) and island site

^d^Average across 11 chromosome-3 loci from [21], only comparison within *An. gambiae* (S-form) between Libreville (mainland) and island site

^e^Global *F*_ST_ for 6 loci between 7 island and 6 mainland villages, from [24]

^f^Average across 17 loci from [25], only comparisons between island versus mainland sites; smaller value in parenthesis is average across 13 loci after removal of 4 in neighborhood of inversions (Table 6 of [25])

^g^Based on whole genome sequences from [9], comparisons limited to *An. gambiae* (S-form) excluding Kenya

^h^Based on 52- and 31-SNP arrays from [20]

## Conclusions

The most recent assessment of malaria control in Africa using currently available tools is not all good news [1]. Hard-won gains against malaria brought about in part by massive distribution of bed nets are now stalled or eroding in some countries, owing to uneven distribution, insecticide resistance, vector behavioral changes, and other difficulties, emphasizing the need for new control measures that could include genetically modified mosquitoes. In accordance with recent guidelines [27], the first open field releases of genetically modified mosquitoes should be tested on physical or ecological islands. We have shown that *An. gambiae* populations on the Ssese Islands, which are within 60 km from mainland Uganda, are genetically differentiated even if their isolation from the mainland may not be absolute. Smaller vector population sizes on these islands, minimal complexity of vector species present [only *An. gambiae* (*s.s*.)], relatively small size and flat terrain of the islands, and logistical ease of access from the mainland, may all be considered advantages in the initial testing phases. Unfortunately, even by Ugandan standards, there is also a heavy burden of malaria on these islands. Compared to urban areas of Uganda, where malaria prevalence in children 6–59 months is 11.5%, malaria prevalence in rural areas is considerably higher, reaching nearly 35% on the mainland and 44% in the islands of Lake Victoria [54]. As such, the Ssese Islands may be prime candidates for the testing of gene-drive and other genetically modified *An. gambiae* mosquitoes.

## Abbreviations

AMOVA: analysis of molecular variance
DNA: deoxyribonucleic acid
LD: linkage disequilibrium
MG: protein coding mitogenome
mtDNA: mitochondrial DNA
*nad*5: NADH dehydrogenase subunit 5
SNP: single nucleotide polymorphism
